# Olive oil protects against progression of heart failure by inhibiting remodeling of heart subsequent to myocardial infarction in rats

**DOI:** 10.14814/phy2.15379

**Published:** 2022-08-08

**Authors:** Abd Al‐Rahman Al‐Shudiefat, Ana Ludke, Akshi Malik, Davinder S. Jassal, Ashim K. Bagchi, Pawan K. Singal

**Affiliations:** ^1^ Department of Physiology and Pathophysiology, Rady Faculty of Health Sciences, Institute of Cardiovascular Sciences St. Boniface Hospital Albrechtsen Research Centre, University of Manitoba Winnipeg Manitoba Canada; ^2^ Department of Medical Laboratory Sciences The Hashemite University Zarqa Jordan; ^3^ Section of Cardiology, Department of Internal Medicine, Rady Faculty of Health Sciences University of Manitoba Winnipeg Manitoba Canada; ^4^ Department of Internal Medicine, Cardiology Division University of Arkansas for Medical Sciences and the Central Arkansas Veterans Healthcare System Little Rock Arkansas USA

**Keywords:** heart failure, myocardial infarction, olive oil, oxidative stress, tumor necrosis factor‐α

## Abstract

We examined the beneficial effects of olive oil against heart failure post‐myocardial infarction (PMI), induced by coronary artery ligation in rats. Animals were divided into sham and ligated groups and fed either regular chow, olive oil (10% wt/wt), or corn oil (10% wt/wt) and were followed up to 16 weeks. On the echocardiography at 3 days (PMI), in the ligated regular chow (LRC), ligated olive oil (LOO), and ligated corn oil (LCO) left ventricular ejection fraction (LVEF) decrease was 12.14%, 16.42%, and 17.53% from the baseline, respectively. However, only LOO group improved LVEF significantly at 16 weeks PMI and became comparable with all sham groups. Both scar formation and collagen deposition at 16 weeks PMI were less pronounced in the LOO group. Myocardial TNF‐α level at 4 weeks of PMI increased by 176%, 11%, and 181% in the LRC, LOO, and LCO groups, respectively. Plasma TNF‐α levels in LOO were significantly lower than LRC group after 4 weeks of PMI. Myocardial redox ratio (reduced glutathione/oxidized glutathione) decreased at 4 weeks PMI by 44.4%, 16.4%, and 36.9% in the LRC, LOO, and LCO groups, respectively, compared to the baseline. These changes in the redox ratio at 16 weeks PMI were further exacerbated in the LRC and LCO groups. Lipid hydroperoxides formation increased at 4 weeks PMI by 137.4%, 14.6%, and 97.1% in the LRC, LOO, and LCO groups, respectively. Since coronary artery ligation decreased left ventricular ejection fraction, increased myocardial TNF‐α and oxidative stress, and since olive oil was able to inhibit these effects, it is proposed that dietary olive oil modulates cardiac remodeling and heart failure subsequent to myocardial infarction.

## Graphical abstract

Representative section of the left ventricle wall of the heart at 4 weeks post myocardial infarction. The sections were stained with Masson’s Trichrome: A) Sham Regular Chow, B) Ligated Regular Chow, C) Sham Olive Oil, D) Ligated Olive Oil, E) Sham Corn Oil, and F) Ligated Corn Oil. Fibrosis is shown here in blue color. Fibrosis is pointed by black arrow. Magnification bar shown in black is 200 µm.

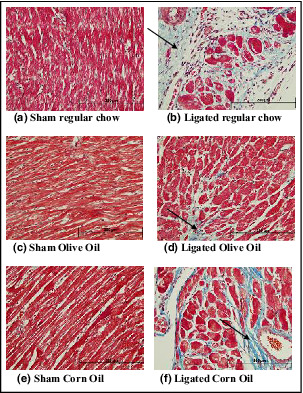

## 1 INTRODUCTION

Cardiovascular disease (CVD) is the leading cause of loss of human life worldwide with an estimated 17.9 million deaths annually (WHO, 2021). The broad term CVD includes: ischemic heart disease, stroke, arrhythmia, cardiomyopathies, congenital heart disease, endocarditis, and valvular heart disease (GBD, 2017; CDC, 2021). The prevalence of ischemic heart disease globally in 2019 was around 40% of all CVD or about 197 million (Roth et al., 2020). It is estimated that 90% of ischemic heart disease could be prevented by controlling risk factors including smoking, obesity, high cholesterol, high blood pressure, unhealthy diet, and a sedentary life (McGill Jr et al., 2008). Myocardial damage due to ischemia or myocardial infarction (MI) causes an increase in oxidative stress and initiates an inflammatory response as well as production of different cytokines including tumor necrosis factor α (TNF‐α) (Kaur et al., 2009; Savic‐Radojevic et al., 2013). Under chronic conditions, these changes contribute to cardiovascular remodeling and heart failure (Bagchi et al., 2017; Zimmer et al., 2020). Levels of soluble TNF‐α receptor 1 are associated positively with the infarct size and left ventricular dysfunction in patients with ST‐elevated myocardial infarction (STEMI) (Nilsson et al., 2013). Therefore, it is essential to prevent or at least slowdown the progress of adverse cardiovascular remodeling before it leads to left ventricular systolic dysfunction and thus heart failure.

Based on 12 different clinical trials, the US Food and Drug Administration (FDA) suggested that a consumption of 1½ tablespoons (20 g) of olive oil every day decrease the risk of coronary heart disease (FDA, 2017; Ghanbari et al., 2012). A recent study on 7447 participants concluded that in the group supplemented with Mediterranean diet, containing extra virgin olive oil, was at a lower risk for developing major cardiovascular events compared to the control group (Estruch et al., 2018). Many other previous studies have reported that the protective effect of olive oil and its major component oleic acid in cardiovascular disease may derive from its antioxidant (Al‐Shudiefat et al., 2013; Fitó et al., 2005), antiapoptotic (Chiang & Chao, 2018; Elgebaly et al., 2018), inotropic (Asoom & Al‐Hariri, 2019; Javidanpour et al., 2018; Loewi, 1955; Pi & Walker, 2000), antihypertensive (Alcaide‐Hidalgo et al., 2020; Massaro et al., 2020), antiatherogenic (Visioli & Galli, 2001; Yanai, 2019), anti‐inflammatory (Fernandes et al., 2020; Santangelo et al., 2018), and a reduction in TNF‐α (Papageorgiou et al., 2011) effects. However, studies are required to show its use in ameliorating fibrosis and inflammation in later stages of MI.

In a study using isolated cardiomyocytes, we reported that TNF‐α‐induced oxidative stress as well as increased expression of apoptotic proteins and cell death were mitigated by oleic acid (Al‐Shudiefat et al., 2013). The present study was undertaken to examine any beneficial effect of olive oil against heart remodeling and failure subsequent to myocardial infarction due to coronary ligation in rats. In order to have a control for the caloric intake with the olive oil, rats fed with the corn oil were also used.

## 2 METHODS

### 2.1 Animal model and study groups

**FIGURE 1:**
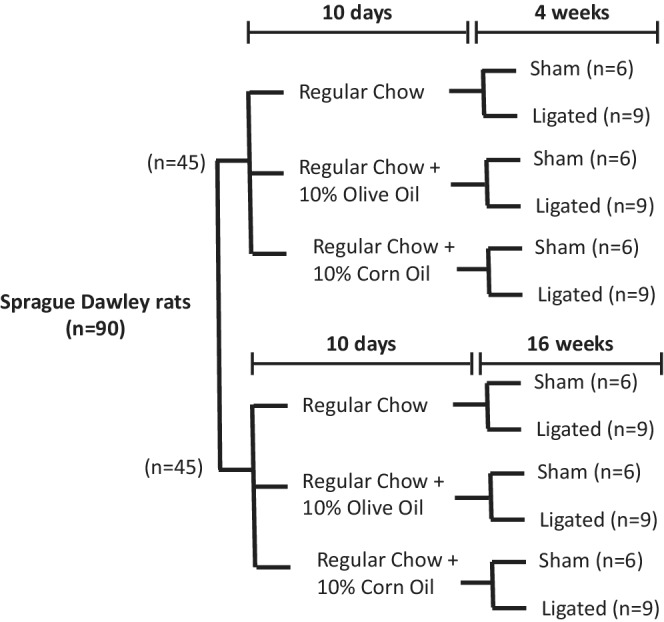
Animal groupings in different diets and sham as well as coronary artery ligation surgeries.

All the experimental work was done according to procedures approved by the University of Manitoba Animal Care Committee, and in line with guidelines of the Canadian Animal Care Committee. Male Sprague–Dawley (SD) (*N* = 90) rats weighing 100 ± 10 g were acclimatized for several days. The animals were divided into three major groups and maintained on different diets (Figure 1): Group 1) regular chow; group 2) regular chow containing 10% w/w olive oil; and group 3) regular chow containing 10% w/w corn oil. In each group, animals were either sham‐operated (6 rats) or coronary ligated (9 rats) and were followed for 16 weeks post‐surgery. Myocardial infarction was done by ligating the left descending coronary artery according to method described previously (Khaper & Singal, 2001). Briefly, rats were anesthetized with 2% isoflurane and the chest hair over the heart region was shaved and disinfected with 3% of hydrogen peroxide and 2% of iodine. About 1–1.5 cm incision was made in the left fifth intercoastal space, parallel to the sternum, and the chest was opened carefully between ribs 5th and 6th by a small retractor to avoid cutting any blood vessel and to visualize the heart. The left coronary artery was determined by appropriate light and gentle pressure by a cotton swap wetted with sterile normal saline. The left coronary artery was tied with a 6–0 monofilament suture. The sham‐operated animals were subjected to the same procedure but the suture around coronary artery was not tied. The chest was closed after aspirating the air.

### 2.2 Special diet preparation

The regular chow (Prolab RMH 3000 5p00) in a pellet form for the regular chow group, and in a powdered form for the other two groups were purchased from Ren's Pet Depot (Guelph, Canada). An antioxidant TBHQ (0.02%) was added to olive and corn oil stocks to prevent rancidity and preserve quality as well as freshness of the diet as recommended by the American Institute of Nutrition (Bieri, 1980; Farhoosh et al., 2012; Fritsche & Johnston, 1988). Commercially available Bertolli Extra Virgin Olive oil was mixed 10%by weight with regular chow powder with 37.5% water using Hobart mixer (Canada) for 3–5 min to make homogenous dough. The same process was done for corn oil. This special food was prepared every 10 days to ensure its freshness. The diet was served in hoppers and the remaining diet was weighed to determine rate of consumption for each singly housed rat.

### 2.3 Murine echocardiography (Echo)

Transthoracic Echo was done on all animals at baseline, 3 days, 2 weeks, 4 weeks, and 16 weeks post‐surgery as described previously (Jassal et al., 2009; Kaur et al., 2006). The parameters measured included: heart rate (HR), left ventricular end diastolic diameter (LVEDd), LV end systolic diameter (LVEDs), and LV ejection fraction (LVEF). Briefly, rats were anesthetized with isoflurane (2%) in a chamber and then mask (1.5%–2% isoflurane) was used for maintaining anesthesia and kept on 37°C temperature regulator pad. The precordial chest hairs were removed and the area was cleaned with iodine and a thin layer of gel was applied. After the pinch confirmation that animal is anesthetized, echocardiography was performed using a 13 MHz probe (Vivid 7, GE Medical Systems). Left ventricular ejection fraction (LVEF) was determined manually using left end diastolic volumes (LVEDV) and left ventricular end systolic volumes (LVESV).

### 2.4 Histological studies

Freshly fixed tissue in buffered Formalin‐Ca was dehydrated by gradually passing through increasing concentrations of alcohol (Randhawa & Singal, 1992). Tissues were dried of alcohol by passing through 100% xylene and then molded into paraffin blocks. Thin tissue sections (5 μM) were cut, stained using Masson's trichrome stain (Randhawa & Singal, 1992) and examined under an Olympus DP70 (Olympus America) microscope to visualize cardiac structural changes and fibrosis.

### 2.5 Biochemical studies

Biochemical parameters were analyzed in the plasma as well as the viable portion of the heart from all groups which included TNF‐α from the plasma and the myocardial tissue, as well as oxidative stress (reduced and oxidized glutathione and lipid hydroperoxides) in the myocardial tissue. For histological as well as biochemical studies, we used viable portion of the left ventricle from the MI hearts and the corresponding myocardial tissue area from its control hearts.

### 2.6 Measuring of myocardial and plasma TNF‐α

Hearts were washed with PBS and scar tissue was removed. The left ventricle was cut using sterile scissor, snap‐frozen in liquid nitrogen, and stored in −70°C freezer. For TNF‐α analysis, 0.1 g of the tissue was washed in PBS and cut into small pieces for homogenization in 1 ml of radioimmunoprecipitation assay buffer (RIPA) containing protease inhibitors and phosphatase inhibitors for 5 s using a PT‐3000 polytron homogenizer (Brinkmann Instruments, Inc). During homogenization, tubes were kept on ice to prevent loss of TNF‐α protein. The homogenate was centrifuged at 10,000*g* for 25 min at 4°C. The supernatant was carefully transferred to another Eppendorf tube and assayed for myocardial TNF‐α using ELISA kit (R & D Systems, Minneapolis, MN) as previously described (Kaur et al., 2006; Torre‐Amione et al., 1999). Briefly, a 50 μl aliquot (50–80 μg protein) of the sample was added to the assay buffer in the kit and analyzed in duplicates with proper control and standards, according to manufacturer's instructions. For plasma TNF‐α, a 50 μl of plasma samples of twofold dilution was assayed similar to myocardial TNF‐α, the final concentration was multiplied by two to compensate for the twofold dilution. Samples were read at 450 nm and the final concentration of the TNF‐α was expressed as picogram per milligram (pg/mg) for the heart and picogram per milliliter (pg/ml) for the plasma.

### 2.7 Oxidative stress measurement

Both glutathione levels and lipid hydroperoxides were analyzed in the myocardium for assessment of oxidative stress.

### 2.8 Glutathione (GHS/GSSG) levels

Myocardial reduced glutathione (GSH) and its oxidized form (GSSG) were measured in all groups using a commercially available kit (Cayman Company, USA). The assay depends on recycling of GSH reductase enzyme (Baker et al., 1990). A total of 0.1 g of left ventricular tissue was homogenized with 1 ml RIPA buffer and centrifuged at 10,000*g* for 15 minutes at 4°C. The supernatant was taken on ice and deproteinized using 4 M triethanolamine and 10% metaphosphoric acid. Fifty microliters of each sample along with proper standards was analyzed in duplicates according to the manufacturer’s instructions. The sulfhydryl group of GSH reacts with 5, 5′‐dithio‐*bis*‐2‐(nitrobenzoic acid (DTNB), Ellman's reagent and results in a yellow substance 5‐thio‐2‐nitrobenzoic acid (TNB). Then samples were read at 405 nm using spectrophotometer to determine GSH. Also, the assay is capable of measuring GSSG only by derivatizing GSH using 2‐vinylpyridine after deproteinization of samples by 4 M triethanolamine and 10% metaphosphoric acid, then the sample were read at 405 nm. GSH was calculated by subtracting GSSG concentration from the total glutathione (GSH + GSSG). Both GSH and GSSG were expressed as μmol/g wet tissue weight. Redox ratio was measured by dividing GSH over GSSG.

### 2.9 Study of lipid hydroperoxides

Lipid hydroperoxides were assessed in all groups using a commercially available kit (LPO‐CC assay kit from Kamiya Biomedical Company in Seattle, USA) as described previously (Khaper & Singal, 2001). Myocardial tissue (0.1 g) was washed with PBS and cut into small pieces, and then homogenized in 1.5 ml of chloroform: methanol (2:1 v/v) solution on ice for 5 s, five times using PT‐3000 polytron homogenizer (Brinkmann Instruments). The mixture was then centrifuged at 3000*g* for 5 min, the supernatant was removed and the chloroform layer containing lipid was evaporated using nitrogen gas for about 20 mins until dry. Lipid residues were dissolved in 100 μl isopropanol, and 15 μl of samples was assessed in duplicates with proper standards according to the manufacturer's instructions. The reaction mixture uses a derivative of methylene blue which is cleaved by lipid hydroperoxides to yield methylene blue then measured at 675 nm by spectrophotometer. Standards were used to evaluate the concentration of lipid hydroperoxides and expressed as nmol/g myocardial tissue.

### 2.10 Protein estimation and statistical analysis

Total protein was assessed using the Bradford method (Bradford, 1976). Briefly, 10 μl of diluted samples and six standards of bovine albumin (0.05, 0.1, 0.2, 0.3, 0.4, and 0.5 mg/mL) were assayed in triplicates on a flat bottom microtiter plate and 200 μl of 1 X protein assay dye (Bio‐Rad) was added to each well and mixed very well. After 5 min of incubation, microtiter plate was read at an ELISA reader at 600 nm. A standard curve was used to estimate the protein in the sample after taking the average of triplicates.

All experiments were done in duplicates and triplicates for each treatment group and repeated five times (*N* = 5). Data are expressed as the mean ± SEM. Groups were compared by one‐way analysis of variance (ANOVA), and Bonferroni's test was performed to identify differences between groups. *p* value of ≤0.05 was considered significant.

## 3 RESULTS

### 3.1 General

Sham control and ligated animals were regularly monitored for their weight and general wellbeing. The mortality rate for the ligated animals was about 13% and most of these deaths were within 24 h after surgery. There was a gradual increase in the animal weight ranging from 184.9 ± 10.4 g in the first week to 750 ± 176.5 g in the 16 weeks post‐myocardial infarction (PMI). There was no significant difference in the body weight of sham regular chow and ligated regular chow, sham olive oil and ligated olive oil, and sham corn oil and ligated corn oil rats at any of the time points in the study. Animals 16 weeks PMI appeared more stressed as indicated by the presence of porphyrin stain and ruffled hair. The consumption of special diet (olive vs. corn oils) for both sham and ligated groups increased with time from the first week until the fifth week and became steady thereafter. The special diet consumption ranged from 23.03 ± 1.56 g/day (wet weight) or 14.43 ± 0.98 g/day (dry weight) in the first week to 59.76 ± 7.67 g/day (wet weight) or 37.35 ± 4.79 g/day (dry weight) in the fifth week and afterward. Olive oil being 10% by wet weight of the regular rat chow, its consumption ranged from 2.30 ± 0.156 to 5.97 ± 0.77 per day/animal.

### 3.2 Histology

**FIGURE 2:**
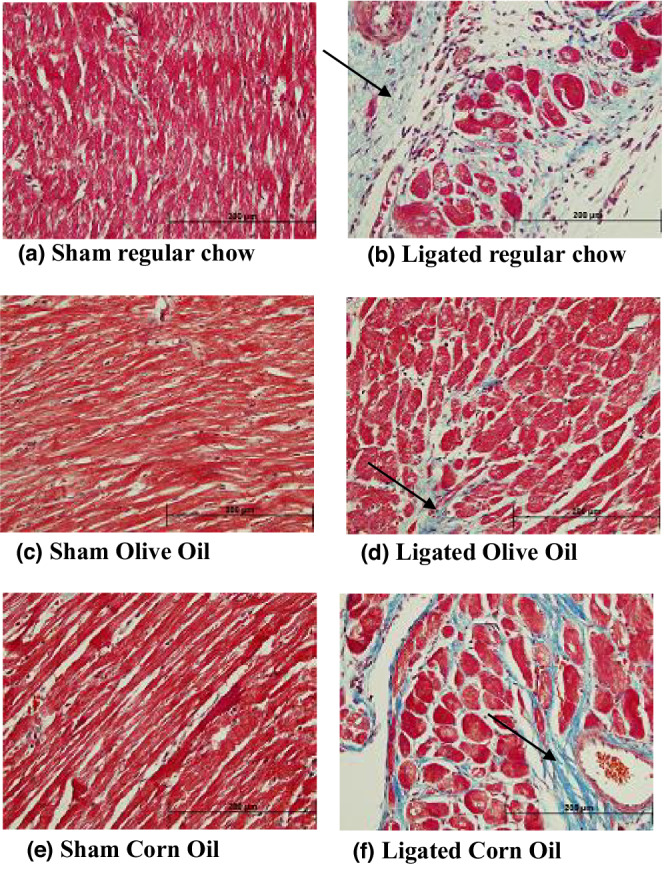
Representative section of the left ventricle wall of the heart at 4 weeks of PMI. The sections were stained with Masson's trichrome: (a) sham regular chow, (b) ligated regular chow, (c) sham olive oil, (d) ligated olive oil, (e) sham corn oil, and (f) ligated corn oil. Fibrosis is shown here in blue color. Fibrosis is pointed by black arrow. The magnification bar shown in black is 200 μm.

**FIGURE 3:**
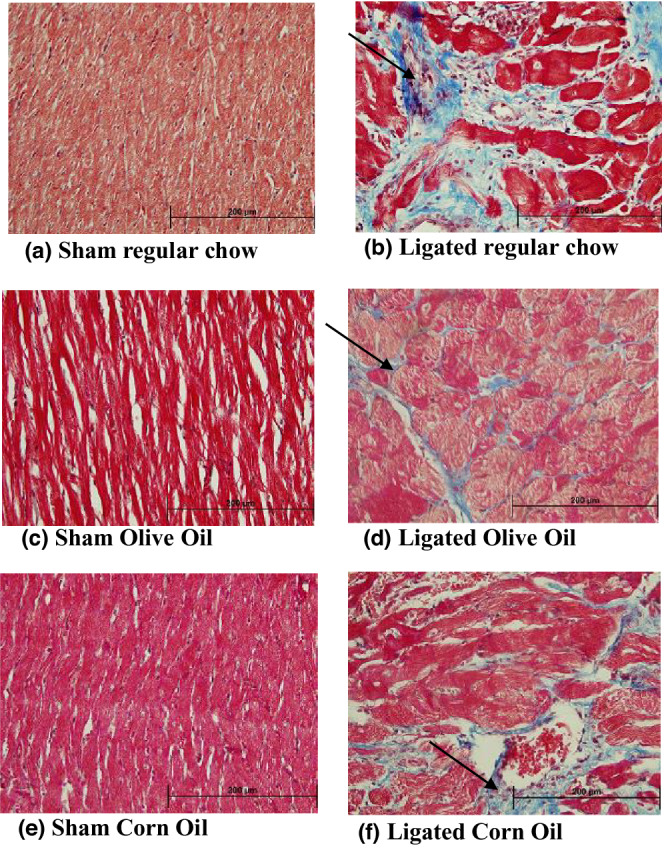
Representative section of the left ventricle wall of the heart at 16 weeks PMI. The sections were stained with Masson's trichrome: (a) sham regular chow, (b) ligated regular chow, (c) sham olive oil, (d) ligated olive oil, (e) sham corn oil, and (f) ligated corn oil. Fibrosis is shown here in blue color and pointed by black arrow. The magnification bar shown in black is 200 μm.

Ligation resulted in the formation of scar as well as hypertrophy of the left ventricular wall and septum. Whole heart hypertrophy was apparent at 4 and 16 weeks PMI in the ligated regular chow as well as in the ligated corn oil groups (data not shown). At 4 weeks PMI, the scar area was principally fibrotic and stained blue, whereas the left ventricle wall as well as septum areas remote from the scar showed cardiomyocyte hypertrophy (Figure 2b,d,f). In the ligated regular chow and corn oil, there was a visual trend of more fibrosis and relatively less myocardial tissue (Figure 2b and f) as compared to ligated olive oil group (Figure 2d). At 16 weeks PMI, the scar formation as well as resulting collagen deposition were more pronounced in both the ligated regular chow (Figure 3b) and ligated corn oil group (Figure 3f). In the olive oil group, there was a trend of less deposition of the collagen (Figure 3d).

### 3.3 Hemodynamic and echocardiographic data

**FIGURE 4:**
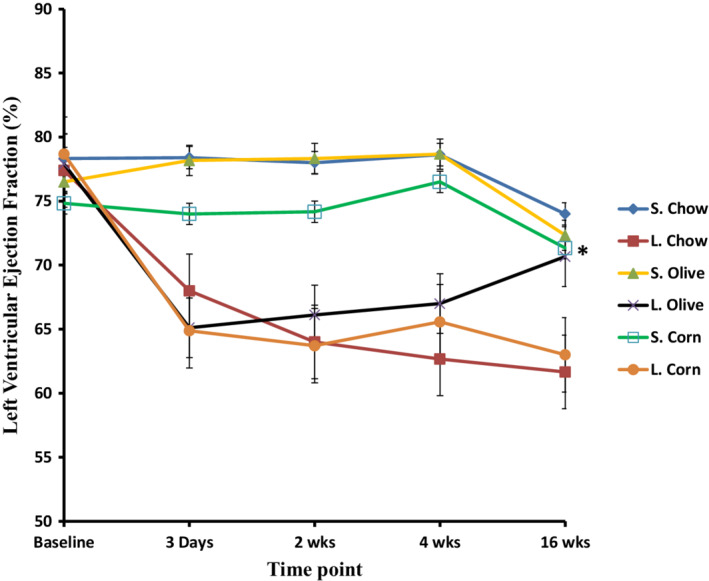
Left ventricular ejection fraction (LVEF) of sham (S) and ligated (L) animals for all groups at different time points of coronary artery ligation. Notice the improvement and restoration of LVEF in the ligated olive oil group (black line) compared to other ligated groups. Data are presented as mean ± SEM of six animals for each group. Two‐way ANOVA analysis was done. *) significant (*p* < 0.05) from the other ligated groups.

**FIGURE 5:**
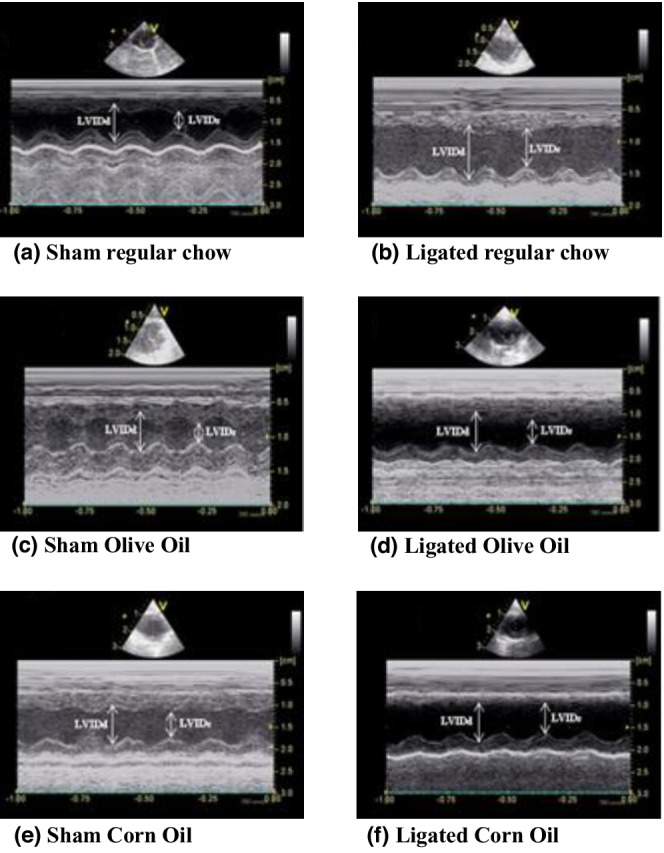
Representative echocardiography images showing left ventricular internal dimension at diastole (LVIDd) and systole (LVIDs), pointed by white arrows in all groups at 16 weeks PMI. (a) Sham regular chow, (b) ligated regular chow, (c) sham olive oil, (d) ligated olive oil, (e) sham corn oil, and (f) ligated corn oil.

All three sham controls showed no change in their systolic or diastolic pressures (Figures 4 and 5). There was no difference in the LVEF among all sham animals throughout the study period (Figures 4 and 5). Whereas, after 3 days PMI, ligated animals in all three groups showed a significant drop in LVEF ranging between 12.14% and 17.5% from the baseline. At 16 weeks, coronary ligation caused a significant increase in left ventricular end diastolic as well as systolic diameters in the ligated regular chow group and corn oil group (Figure 5b and f compared with 5a and 5d). However, only the ligated olive group began to show improvement at 2 weeks and at 16 weeks PMI, it became comparable with its sham group (Figures 4 and 5). Ligated corn oil and ligated regular chow groups showed no such recovery at 16 weeks (Figures 4 and 5). These data indicate that changes in cardiac remodeling seen in histological studies can be contributing to the improvement in cardiac function post‐MI when olive oil is supplemented in the diet.

### 3.4 Myocardial and plasma TNF‐α

**FIGURE 6:**
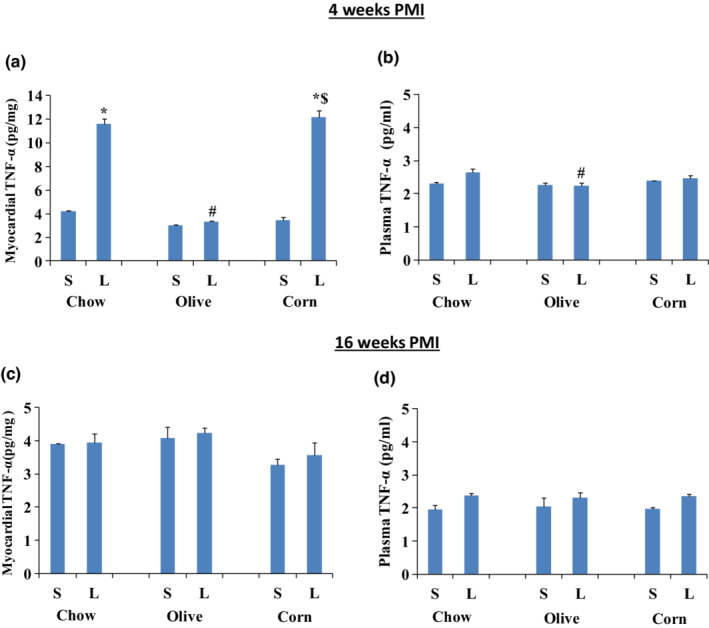
Myocardial and plasma TNF‐α protein levels in sham (S) and ligated (L) animals after 4 and 16 weeks PMI. (a) Myocardial and (b) plasma TNF‐α protein levels at 4 weeks PMI; (c) myocardial, and (d) plasma TNF‐α protein levels at 16 weeks PMI. TNF‐α from myocardial tissue is expressed as pg/mg of protein, whereas for plasma it is pg/ml of the plasma. Data are presented as mean ± SEM of six animals for each group. Two‐way ANOVA analysis was done. *) significant from its own sham, #) significant from ligated regular chow, $) significant from ligated olive oil. In all groups *p* < 0.05. The scale in Figure 6(a) is different from that in Figures 6(b, c and d).

TNF‐α protein levels were determined in myocardial tissues as well as in plasma in all groups at both 4 and 16 weeks PMI. Myocardial and plasma TNF‐α of sham groups at 4 and 16 weeks PMI were within the range of 2.98 ± 0.08 pg/mg to 4.23 ± 0.15 pg/mg (Figure 6). At 4 weeks PMI, there was a significant increase (176%) in the myocardial TNF‐α level in the ligated regular chow group (Figure 6a) from 4.2 ± 0.06 pg/mg in the sham control to 11.58 ± 0.47 pg/mg in the ligated group. There was no change in TNF‐α level in the ligated olive oil compared to its sham or sham regular chow, but ligated olive oil group showed significantly lower levels of TNF‐α compared to ligated regular chow and ligated corn oil groups. There was a significant increase (251%) in the TNF‐α level from 3.46 ± 0.27 pg/mg in sham to 12.16 ± 0.55 pg/mg in ligated corn oil (Figure 6a). At 16 weeks PMI, myocardial TNF‐α protein levels were comparable and within the normal range for all groups (Figure 6c). At 4 weeks PMI, the plasma level of TNF‐α protein was significantly lower in ligated olive oil group, compared to ligated regular chow (Figure 6b). At 16 weeks PMI, the plasma level of TNF‐α protein was comparable with no significant change in any of the groups (Figure 6d).

### 3.5 Oxidative stress assessment

Myocardial oxidative stress levels were assessed by measuring the ratio of reduced glutathione (GSH) to oxidized glutathione (GSSG) as well as the levels of lipid hydroperoxide:

#### 3.5.1 Redox ratio (GSH/GSSG)

**FIGURE 7:**
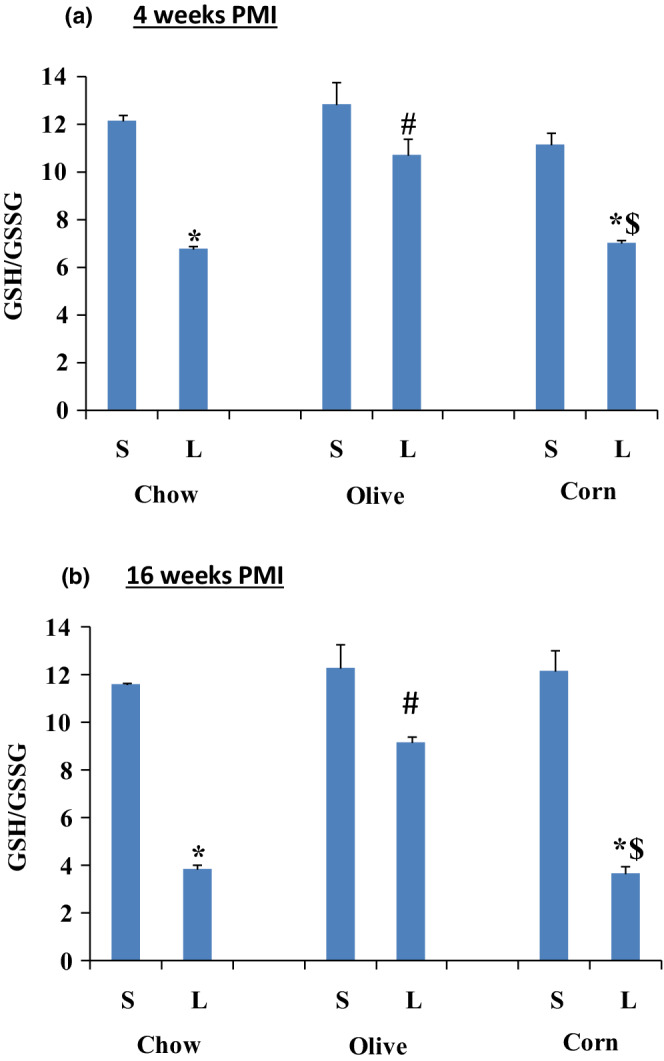
Myocardial redox ratio (GSH/GSSG) for sham (S) and ligated (L) animals after 4 and 16 weeks PMI. A) GSH/GSSG after 4 weeks PMI; and B) GSH/GSSG after 16 weeks PMI. Data are presented as mean ± SEM from six animals for each group. Two‐way ANOVA analysis was done. *) significant from its own sham, #) significant from ligated regular chow, and $) significant from ligated olive oil. In all groups *p* < 0.05.

Myocardial reduced (GSH) and oxidize glutathione (GSSG) levels were measured in all sham and ligated groups at 4 and 16 weeks PMI and the redox ratios (GSH/GSSG)—an indication of oxidative stress, were determined (Figure 7). Redox ratio decreased significantly in all ligated animals except ligated olive oil at 4 weeks PMI (Figure 7). At 4 weeks PMI, redox ratio decreased by 44.4% in the ligated regular chow and only 16.4% in the ligated olive oil, and 36.9% in the ligated corn oil (Figure 7). At 16 weeks PMI, the decrease was 67.2% in the ligated regular chow, 25.2% in the ligated olive oil, and 70% in the ligated corn oil (Figure 7).

#### 3.5.2 Lipid hydroperoxides

**FIGURE 8:**
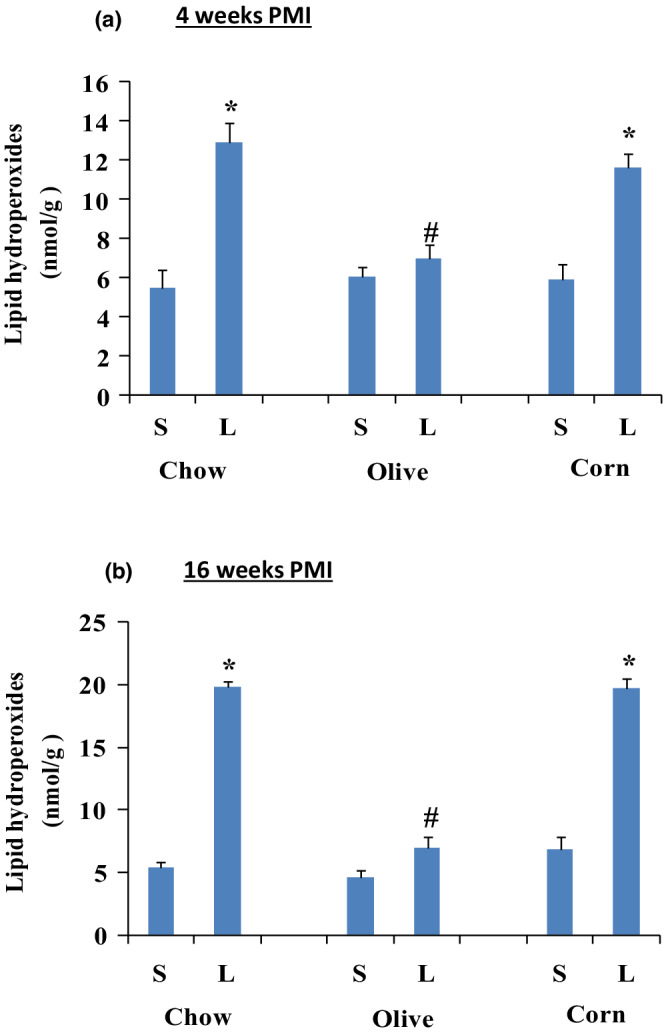
Myocardial, lipid hydroperoxide levels are expressed as per mg of myocardial tissue in sham (S) and ligated (L) animals after 4 (a) and 16 (b) weeks PMI. Data are presented as mean ± SEM from six animals for each group . Two‐way ANOVA analysis was done. *) significant from its own sham, #) significant from the ligated corn oil. In all groups, *p* < 0.05.

Coronary artery ligation increased lipid hydroperoxides levels in all ligated regular chow and corn oil as compared to their respected sham groups but not in the ligated olive oil group (Figure 8). At 4 weeks PMI, lipid hydroperoxides levels increased 137.43% in the ligated regular chow, 14.68% in the ligated olive oil, and 97.1% in the ligated corn oil (Figure 8). At 16 weeks PMI, lipid hydroperoxides increased 272% in the ligated regular chow, 32% in the ligated olive oil, and 187% in the ligated corn oil group (Figure 8).

## 4 DISCUSSION

In an earlier study on isolated cardiomyocytes, we have reported that TNF‐α exposure caused an increase in oxidative stress, myocyte dysfunction, and cell death (Al‐Shudiefat et al., 2013). These effects in cardiomyocytes were mitigated by oleic acid—a major component of olive oil (Al‐Shudiefat et al., 2013). In the SOLVD (study of left ventricular dysfunction) trial, increased TNF‐α levels in the blood were correlated with a severity of heart failure (Torre‐Amione et al., 1996). It is also known that in MI subsequent to reduced or stoppage of coronary flow, there is an elaboration of TNF‐α in the early stages which may play an important role in cardiac remodeling and heart failure (Kaur et al., 2009; Nian et al., 2004). In the present in vivo study, using coronary ligated MI animals, a diet supplemented with olive oil protected against cardiac remodeling, increase in TNF‐α and structure–function changes. In order to rule out that this beneficial effect of olive oil was not due to the increase in caloric intake, we used corn oil as the control. Since no such beneficial effect was seen in the ligated corn oil group, the protective effect with the olive oil can be attributed to its active components, particularly oleic acid (Al‐Shudiefat et al., 2013). It is thus reasonable to assume that olive oil alone protected against left ventricular dysfunction in coronary ligated groups which also showed improved ventricular structure and ejection fraction comparable to their sham control.

Coronary artery ligation increased TNF‐α levels in the early stages in the ligated group which have been reported to implicate in left ventricular dysfunction through an increase in oxidative stress (Al‐Shudiefat et al., 2013; Dhingra et al., 2007) and oleic acid—a component of olive oil was able to mitigate these effects (Al‐Shudiefat et al., 2013). This may also involve antioxidant properties of olive oil (De Bruno et al., 2021). TNFα elaboration after MI may also lead to impairment of heart contractility by a direct depression of the shortening of the sarcomeres and dilatation of heart due to the production of nitric oxide as well as by decreasing availability of intracellular calcium (Finkel et al., 1992; Kapadia et al., 1995). In addition to its antioxidant property, the improvement of left ventricular function may also be supported by the reported inotropic effects of olive oil (Asoom & Al‐Hariri, 2019; Javidanpour et al., 2018; Loewi, 1955; Pi & Walker, 2000). Anti‐inflammatory effects of olive oil have been suggested to mitigate acute ozone effects on the cardiovascular system (Tong et al., 2020) and also in arthritis (Mahmoud et al., 2021). In the present study, olive oil reduced oxidative stress in ligated rats was evident from maintaining of the GSH/GSSG ratio as well as lipid hydroperoxides production comparable to that in the olive sham group. A similar type of protection by olive oil has also been reported in multiple sclerosis (Conde et al., 2020).

There are multiple mechanisms put forward for an explanation of the increase in TNF‐α in heart failure (Blick et al., 1987; Kapadia et al., 1998; Rauchhaus et al., 2000). One suggestion is that an under‐perfusion of body tissues due to a decrease in cardiac output allows for an increase in TNF‐α due to mobilization of endotoxins (Rauchhaus et al., 2000). Another possibility is that the myocardial injury during infarction activates the immune system which may result in the elaboration of TNFα (Kapadia et al., 1998). The third possibility is that an early increase in the plasma may represent a myocardial spill‐over effect which elicits a secondary activation of the immune system (Kapadia et al., 1998). Since TNF‐α has a short half‐life in the plasma (Blick et al., 1987), its increase at 4 weeks PMI may suggest a continuous biosynthesis. At any rate in the present study, there was an increase in the cardiac TNF‐α subsequent to MI which was blunted by a diet enriched with olive oil. Therefore, beneficial cardiac effects of dietary olive oil seem to have been correlated with the attenuation of anti‐inflammatory and pro‐oxidative effects in cardiac tissue.

The study suggests that coronary artery ligation results in an increase in inflammatory cytokine TNF‐α as well as an increase in oxidative stress. All these changes led to the deterioration of heart structure/function. Olive oil was able to decrease TNF‐α level, oxidative stress and protected against left ventricular dysfunction in the MI hearts. In fact, these data also suggest that a daily dietary oil consumption may interfere with multiple biological factors associated with cardiac remodeling subsequent to MI.

## AUTHORS’ CONTRIBUTIONS

A.A.A‐S., P.K.S., A.L., and A.K.B. contributed to experimental design. A.A.A‐S., A.L., and A.K.B. performed the experiments. P.K.S. provided resources and materials. A.A.A‐S., P.K.S., D.S.J., A.L., A.M., and A.K.B. contributed to data analysis, writing, editing, and final submission of the manuscript.

## CONFLICT OF INTEREST

There is no conflict of interest and no relationship with industry to disclose.
